# Clinical application of angiotensin receptor blockers in patients with non-alcoholic fatty liver disease: a systematic review and meta-analysis

**DOI:** 10.18632/oncotarget.23816

**Published:** 2018-01-02

**Authors:** Yating Li, Hong Xu, Wenrui Wu, Jianzhong Ye, Daiqiong Fang, Ding Shi, Lanjuan Li

**Affiliations:** ^1^ State Key Laboratory for Diagnosis and Treatment of Infectious Diseases, Collaborative Innovation Center for Diagnosis and Treatment of Infectious Diseases, The First Affiliated Hospital, Zhejiang University, Hangzhou 31003, People’s Republic of China; ^2^ Department of Orthopaedics, Tianjin Medical University General Hospital, Heping District, Tianjin 300052, People’s Republic of China

**Keywords:** NAFLD (non-alcoholic fatty liver disease), ARBs (angiotensin receptor blockers), liver fibrosis, liver inflammation, meta-analysis

## Abstract

**Objective:**

Non-alcoholic fatty liver disease (NAFLD) is one of the most common chronic liver diseases, ranging from simple steatosis to progressive steatohepatitis and cirrhosis. Because of their anti-inflammatory and anti-fibrotic effects, angiotensin receptor blockers (ARBs) are potential therapeutic agents for NAFLD. The present systematic review assessed the effectiveness of ARBs in NAFLD management.

**Results:**

Accounting for data overlap and exclusion criteria, randomized controlled trial -based and single-arm meta-analyses were conducted for four studies with 362 patients and eight studies with 525 patients, respectively. Although alanine aminotransferase levels were not significantly affected by ARB treatment (standardized mean difference 0.20; 95% confidence interval (CI) [−0.04, 0.44]; *P =* 0.10), a fixed-effect model revealed a decreasing trend in alanine transaminase levels. Low-density lipoprotein levels were reduced by ARB treatment (MD 5.21; 95% CI [3.01, 7.40]; *P* < 0.00001), and total cholesterol also decreased in response to ARBs (MD 2.10; 95% CI [−0.37, 4.57]; *P =* 0.10). However, the fibrosis score and NAFLD activity score were not significantly improved by ARB treatment (MD 0.10; 95% CI [−0.58, 0.78]; *P =* 0.77) (MD −0.25; 95% CI [−1.05, 0.55]; *P =* 0.53).

**Materials and Methods:**

Keywords were used to identify studies in PubMed, EMBASE, CENTRAL, Web of Science and CNKI published up to July 31, 2017. Single-arm and RCT-based meta-analyses of the available data were performed using RevMan (version 5.3).

**Conclusions:**

Although ARBs significantly decreased plasma low-density lipoprotein and total cholesterol levels, the current evidence is insufficient to support the efficacy of ARBs in managing fibrosis in NAFLD patients.

## INTRODUCTION

Non-alcoholic fatty liver disease (NAFLD) is one of the most common chronic liver diseases, ranging from simple steatosis to progressive steatohepatitis and cirrhosis [1]. The major risk factors for NAFLD include adiposity, hyperlipidemia, insulin resistance (IR), inflammatory cytokines and oxidative stress responses, type 2 diabetes mellitus (T2DM) and metabolic syndrome (MS) [2]. The main strategy for the treatment of NAFLD is currently based on lifestyle modification and pharmacotherapy targeting hepatic fat accumulation, metabolic stress, oxidative stress, inflammation and modulation of gut microbiota [1, 3].

Recently, the renin-angiotensin-aldosterone system (RAAS), which has a central function in the physiology of blood pressure, was reported to be associated with inflammation and fibrosis in NAFLD [4]. Moreover, renin-angiotensin system blockers (RAS-B), including angiotensin-converting enzyme inhibitors (ACEIs) and angiotensin receptor blockers (ARBs), have been shown to exert protective effects against liver fibrosis [5–8]. These effects are due to suppression of hepatic stellate cell transformation into hepatic myofibroblasts (HMs) in response to elevated expression of pro-inflammatory cytokines [9, 10] and reduced expression of tissue growth factors, angiotensin II type-1 receptor (AT1R), and TGF-β1 [11, 12]. HMs possess a localized renin-angiotensin system (RAS) that continuously produces angiotensin II and stimulates fibrogenesis [13, 14]. A study in rats found that the ARB telmisartan markedly improved hepatic fibrosis and inhibited disease progression, providing evidence that RAS inhibitors are potential therapeutic molecules [15].

ARBs have also been found to regulate hepatic lipid metabolism [16]. Lipid accumulation is reduced in the absence of AT1R, with significant induction of PPAR, and olmesartan inhibits AT1R [8] and promotes hepatic lipid homeostasis, with no impact on PPARc activation. Therefore, AT1R blockade may be efficacious in the treatment of NAFLD or non-alcoholic steatohepatitis (NASH) [16].

Although the functions of ACEIs and ARBs in preventing complications of NAFLD have been widely investigated in mice, clinical data from patients are lacking, [17] and the effectiveness of ARBs in treating NAFLD remains controversial. For example, losartan has positive effects on biochemical variables, hepatic steatosis, inflammation, and serum biomarkers of fibrosis in patients with NASH, [18, 19] but a subsequent study showed no additional benefits of losartan on liver histology when combined with rosiglitazone [20]. Telmisartan and valsartan were reported in separate studies to improve alanine aminotransferase levels and homeostatic model assessment of insulin resistance (HOMA-IR) scores, but only telmisartan reduced NAFLD activity (NAS) and fibrosis (F-S) scores [21]. Therefore, we conducted a systematic review and meta-analysis of clinical randomized controlled trials (RCTs), retrospective studies, and pilot prospective studies in patients with NAFLD.

## MATERIALS AND METHODS

### Search strategy

An extensive electronic search was performed by two independent investigators (YT and XH) for relevant articles published up to July 31, 2017. Databases including PubMed, EMBASE, Cochrane Central Register of Controlled Trials (CENTRAL), Web of Science and China National Knowledge Infrastructure (CNKI) were searched. The searches were performed using medical subject headings (MeSH) combined with the following terms: ‘non-alcoholic fatty liver disease’, ‘angiotensin receptor blockers’, ‘renin-angiotensin-aldosterone system’, and the names of specific ARBs, including valsartan, telmisartan, losartan, irbesartan, azilsartan and olmesartan. The references of relevant review articles were manually searched to identify applicable studies. The publication language was not restricted. Relevant RCTs were manually selected.

### Study selection criteria

Two investigators (YT and XH) independently assessed the eligibility of the identified articles. Titles and abstracts were first screened, and eligible articles were reserved for full-text review. No language restriction was imposed for inclusion. Studies meeting the following inclusion criteria were included: (1) articles including patients with NAFLD, (2) studies on patients treated with ARBs, and (3) studies reporting data on the therapeutic effects of ARBs. The exclusion criteria were as follows: (1) case reports, editorials or review articles; (2) studies involving treatment of NAFLD with strategies other than ARBs; and (3) studies in which the patients’ condition was complicated, such that the therapeutic effect may have been affected by other treatments without any control variate method. Any disagreement or doubts were resolved through discussion to reach a consensus.

### Data extraction

The following data were extracted by the two investigators: year of publication, number of patients, characteristics of patients (age, sex and treatment), location and period of the study, detailed dosage and categories of ARB used for NAFLD, clinical outcomes including F-S, NAS, and HOMA-IR, alanine aminotransferase (ALT), serum aspartate aminotransferase (AST), alkaline phosphatase (ALP), γ-glutamyl transferase (GGT), low-density lipoprotein (LDL), high-density lipoprotein (HDL), total cholesterol (TC) levels, and body mass index (BMI). Data extraction was performed by reading the full articles, tables, figures and interpretations for each included study. Any disagreements were resolved by reaching a consensus.

### Bias & quality assessment

The risk of bias was assessed with the Cochrane Collaboration’s risk of bias tool based on seven domains: random sequence generation, allocation concealment, blinding of participants and outcome assessment, incomplete data, selective reporting, intention to treat analysis, and other sources of bias. Disagreements were resolved by consensus.

All selected articles involving RCTs were assessed for the risk of bias according to Cochrane Handbook for Systematic Reviews of Interventions, which mainly includes seven domains: (1) random sequence generation (selection bias), (2) allocation concealment (selection bias), (3) blinding of participants and personnel (performance bias), (4) blinding of outcome assessment (detection bias), (5) incomplete outcome data (attrition bias), (6) selective reporting (reporting bias), and (7) other bias. The risk of bias in each category was designated as low or high; it was designated as “NA” if the risk was not applicable to the particular study.

### Statistical analysis

RevMan software (version 5.3) was used to pool all appropriate data including NAS and HOMA-IR indices, ALT, AST, GGT, LDL, HDL, TC levels and BMI. Single-arm and RCT-based meta-analyses were performed for each parameter if the data were available. The mean difference (MD) and 95% confidence interval (CI) were calculated. The standardized mean difference (SMD) was employed when different instruments were used to measure the same construct. Heterogeneity of the included studies was assessed and quantified using the I^2^ statistic, with α = 0.05. The fixed-effect model was used if there was no evidence of heterogeneity when I^2^ ≤ 50%; otherwise the random-effect model was employed. Subgroup analysis was performed by grouping similar types of ARBs to reduce significant heterogeneity.

## RESULTS

### Selection of relevant studies

**Figure 1:**
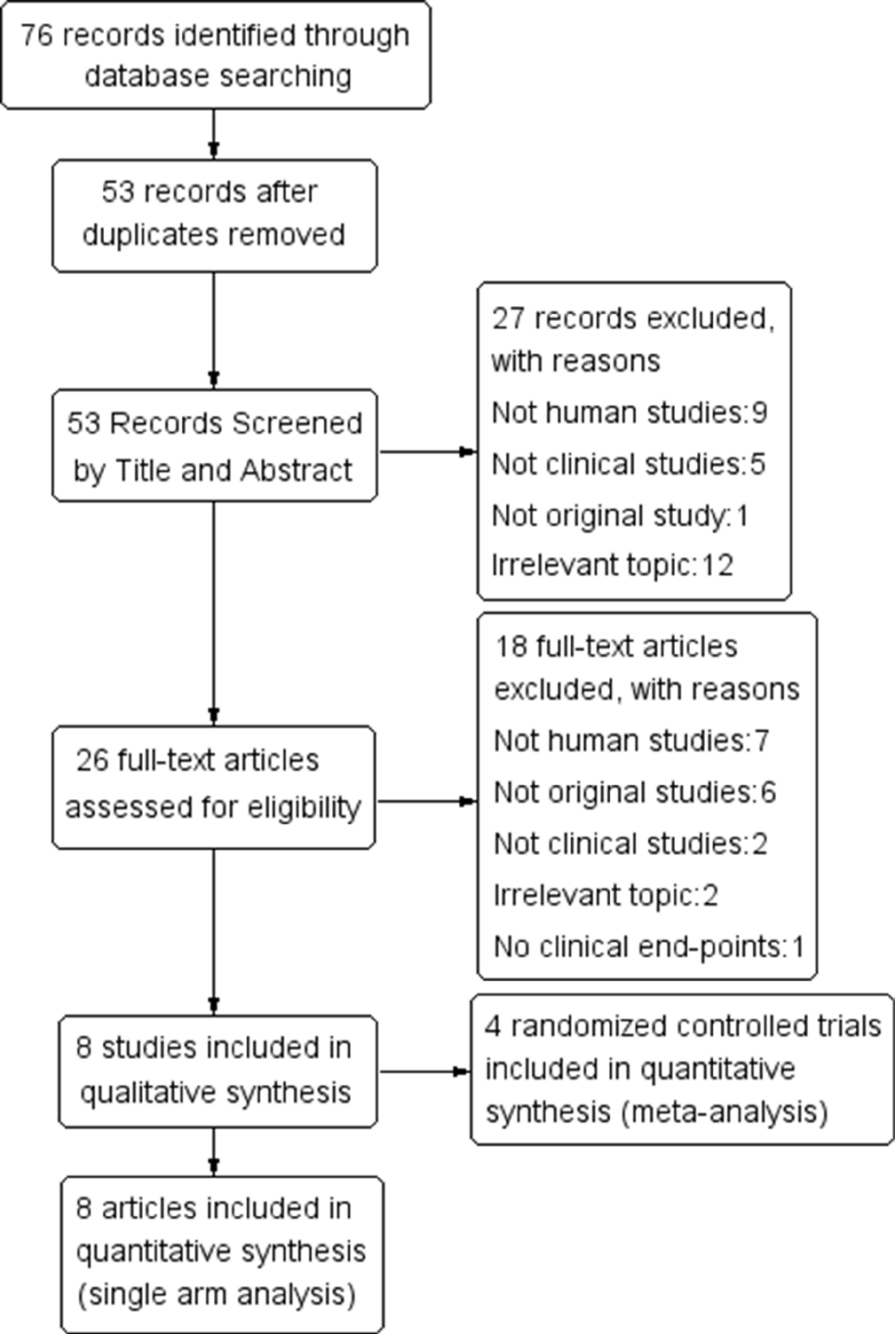
Flowchart of study selection

According to the selection process shown in Figure 1, 76 relevant studies were initially identified. Twenty-three studies were excluded as duplicates, leaving 53 studies for the screening of titles and abstracts. Twenty-six studies were then selected after strict implementation of the inclusion and exclusion criteria. The full texts of the studies were reviewed, which led to the exclusion of 18, including seven non-human studies and six non-original studies, two non-clinical studies, two irrelevant studies, and one study with non-clinical end-points. This process yielded eight articles for quantitative assessment (single-arm meta-analysis) [19–26] and four RCTs for the meta-analysis. The selection process is presented in the form of a flowchart in Figure 1.

### Characteristics of the included studies and analysis

Among the eight included clinical trials, five included patients diagnosed with NASH based on histology; the other three enrolled NAFLD patients. In four studies, losartan was administered at a dose of 50 mg/d or 100 mg/d for at least four months. Telmisartan, valsartan and olmesartan were also used in other studies, with treatment durations ranging from two to 12 months. The study by Alam et al. [22] recommended lifestyle modification for all participants. Patients in the study by Torres et al. [20] were administered an equal dose of rosiglitazone, whereas a placebo was used in the study by McPherson et al. [24]. The summary of the included studies and related outcomes is shown in Table 1. For all pooled RCTs, none of the patients in the control groups received any medication except for the hypertensive patients with NASH in the study by Fogari et al. [23] who received amlodipine.

**Table 1:** Summary of the included studies

Article	Type of study	Patient diagnosis	Number of patients	Schedule (intervention)	Outcome measures	Findings	Follow-up
Alam S 2016 [22]	open-label prospective RCT	NASH	30	Telmisartan 40/80 mg once daily with lifestyle modification/lifestyle modification	1. Biochemical analysis and HOMA-IR 2. Histopathological assessment: NAS, hepatocellular inflammation, hepatocyte ballooning degeneration and fibrosis	1. The improvement in the NAS was significantly higher with telmisartan and lifestyle modification than with lifestyle modification alone. 2. ALT and GGT levels improved but did not differ significantly between the two groups	12 months
Torres DM 2011 [20]	open-label, prospective RCT	NASH	137	Losartan 50 mg once daily/rosiglitazone and metformin	1. Demographic data 2. Biochemical analysis: fasting insulin level, fasting lipid panel, fasting glucose, haemoglobin A1c, and CRP levels, basic metabolic panel, liver function panel and HOMA-IR 3. Histopathology analysis: degree of steatosis, hepatocellular inflammation, hepatocyte ballooning degeneration, NAS	1. No significant improvement was noted for steatosis, hepatocellular inflammation, ballooning or fibrosis between the two groups. 2. Insulin levels and HOMA-IR were significantly improved. 3. Mean body weights increased in both groups.	4 months
Fogari R 2012 [23]	double-blind, RCT	mild to moderate hypertension with hepatic steatosis	150	Losartan 100 mg once daily/amlodipine 10 mg/day	1. Demographic data: weight, BMI, fasting plasma glucose levels, fasting plasma insulin levels, blood pressure, TC, LDL, HDL, TG, leptin, ADN, TNF-a, IL-6, and Hs-CRP levels 2. Ultrasound examination: degree of steatosis, SAT and VAT diameter	1. TC and LDL were decreased in both groups. 2. Decrease in TNF-a, IL-6, and Hs-CRP in ARB group 3. Improvement in VAT and steatosis in patients treated with ARBs.	12 months
McPherson S 2017 [24]	RCT	NASH	45	Losartan 50 mg once daily/ placebo	1. Biochemical and clinical features: AST, ALT, GGT, TG, TC, HDL, and LDL levels 2. Percutaneous liver biopsy: NAS and fibrosis	1. The NAS was not changed over the treatment period in the losartan-treated patients, but it decreased in the placebo-treated patients. 2. All biochemical parameters were similar between baseline and at EOT in both groups.	24 months
Yokohama S 2004 [19]	clinical trial (pilot study)	NASH	7	Losartan 50 mg once daily	1. Laboratory assessment: serum transaminase levels, HOMA-IR, and TGF-β2 levels. Liver biopsy & hepatic fibrosis, including serum hyaluronic acid, type IV collagen and procollagen III N-terminal propeptide levels	1. Serum AST and ALT levels decreased significantly. 2. Plasma markers of hepatic fibrosis were significantly reduced. 3. Lipid profiles, renal function, serum electrolytes, plasma renin activities, serum Ang II levels, and HOMA-IR were unchanged. 4. The degree of lobular steatosis was unaffected by the losartan treatment.	4 months
Enjoji M 2008 [25]	clinical trial (pilot study)	NAFLD	14	Olmesartan 20 mg once daily/ telmisartan 40 mg once daily	HOMA-IR and ALT levels	HOMA-IR and ALT decreased significantly.	6 months
Yuan D 2016 [26]	clinical trial	NAFLD	88	Valsartan 80 mg once daily	1. Clinical blood index: plasma ALT, GGT, TG, TC, GLU, PRA, Ang I and Ang II levels 2. Liver ultrasound	The biochemical index of NAFLD improved.	2 months
Georgescu EF 2009 [21]	RCT	NASH and mild to moderate hypertension	54	Telmisartan 40 mg once daily/valsartan 80 mg once daily	1. Biochemical analyses and histology: FPG, ALT, AST, GGT, bilirubin (B), TC, and TG levels, HOMA-IR 2. Percutaneous liver biopsy: NAS	1. ALT levels were significantly decreased in all patients. This decrease did not differ significantly between group T and group V. 2. HOMA-IR was significantly decreased in all patients at EOT. The mean monthly decrease in HOMA-IR in group T was greater than in group V. 3. The NAS was significantly decreased in all patients at EOT, with a greater decrease in group T than in group V.	20 months

HOMA-IR, homeostatic model assessment of insulin resistance; NAS, non-alcoholic fatty liver disease activity score; Hs-CRP, high-sensitivity C-reactive protein; ADA, adiponectin; AST, aspartate aminotransferase; ALT, alanine aminotransferase, GGT, γ-glutamyl transferase; ALP alkaline phosphatase; CRP, C-reactive protein; SAT, subcutaneous adipose tissue; VAT, visceral adipose tissue; EOT, end of treatment.

**Figure 2:**
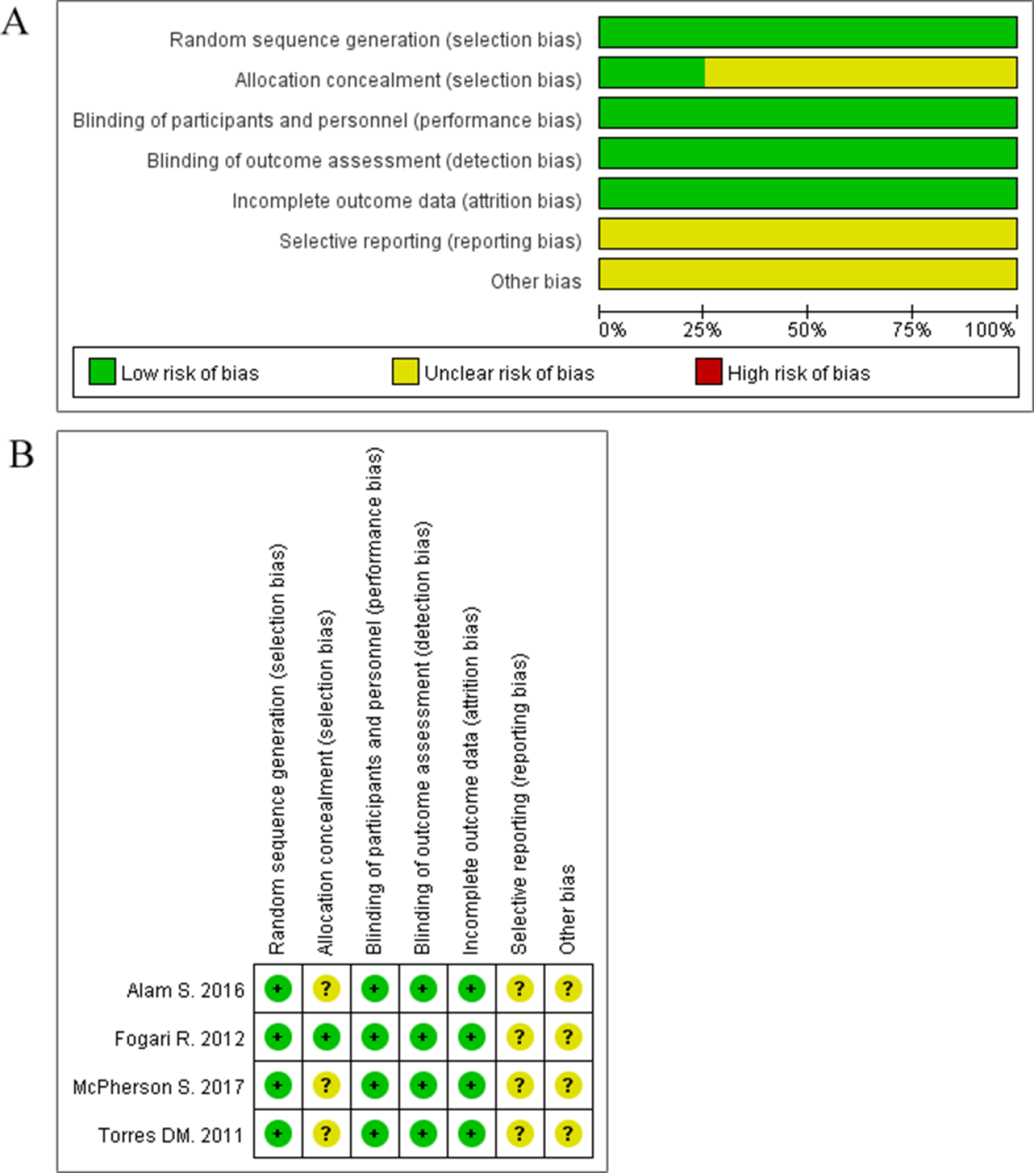
(**A–B**) Summary and graph of risk of bias for all included RCTs.

The quality of the included studies was moderate to high according to the Cochrane Collaboration’s risk of bias tool. The summary of quality assessment domains of the included studies is shown in Figure 2.

### Serum markers of liver damage

**Figure 3:**
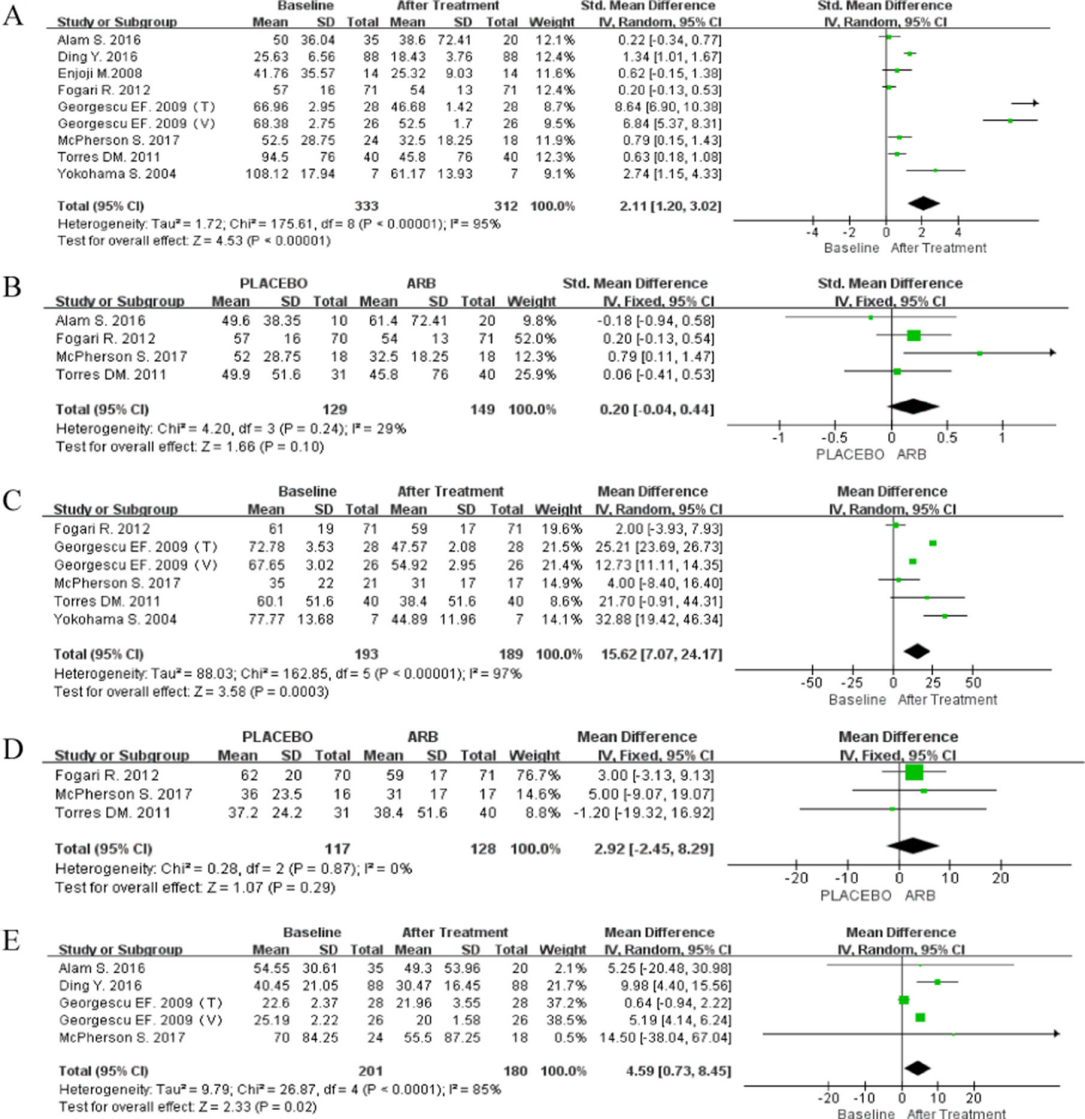
(**A**) Forest plot of the mean differences in the changes in ALT (single-arm meta-analysis). (**B**) Forest plot of mean differences of changes in ALT; IV, inverse variance; CI, confidence interval (RCT-based meta-analysis). (**C**) Forest plot of the mean differences in the changes in AST (single arm meta-analysis). (**D**) Forest plot of the mean differences in the changes in AST (RCT-based meta-analysis). (**E**) Forest plot of the mean differences in the changes in GGT (single arm meta-analysis).

All eight articles reported data on ALT. Single-arm meta-analysis was performed for 333 patients who received ARBs, with available follow-up information for 312 (93.7%). The random-effect model was used because of significant heterogeneity (*P* < 0.00001; I^2^ = 95%), indicating that serum ALT levels were significantly reduced during ARB treatment of NAFLD (SMD 2.11; 95% CI [1.20, 3.02]; *P* < 0.00001; Figure 3A).

An RCT-based meta-analysis was then performed for 278 patients, 149 of whom received ARBs, whereas 139 did not. There was no significant heterogeneity among these studies (*P* = 0.24; I^2^ = 29%). Although the results were not significant (SMD 0.20; 95% CI [–0.04, 0.44]; *P* = 0.10; Figure 3B), a distinct decreasing trend in ALT levels was observed in response to ARBs using the fixed-effect model.

Five trials reported controversial results regarding AST levels in the ARB-treated group. A single-arm meta-analysis was performed with the random-effect model (*P* < 0.00001; I^2^ = 97%), and AST levels were found to be significantly reduced in response to ARB treatment (MD 15.62; 95% CI [7.07, 24.17]; *P* = 0.0003; Figure 3C). The fixed-effect model was then applied for RCT-based meta-analysis (*P* = 0.87; I^2^ = 0%), and the results were similar to those obtained for ALT (MD 2.92; 95% CI -2.45, 8.29); *P* = 0.29; Figure 3D).

Data for GGT levels were pooled from four articles (two RCTs). Because significant heterogeneity was observed across the studies (*P* < 0.0001; I^2^ = 85%), the random-effect model was adopted, which showed that GGT levels were significantly reduced in response to ARBs (MD 4.59; 95% CI [0.73, 8.45]; *P* = 0.02; Figure 3E).

### Lipometabolism and insulin resistance

A significant reduction in HOMA-IR score was reported in five articles, including two RCTs.

**Figure 4:**
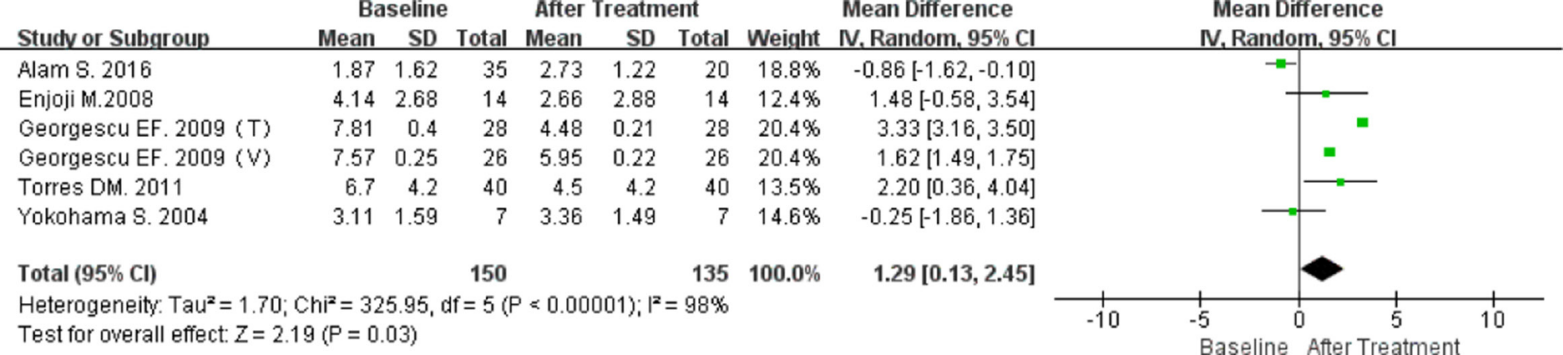
Forest plot of the mean differences in the changes in HOMA-IR (single arm meta-analysis)

A single-arm meta-analysis was performed for 150 patients who received ARBs, with follow-up data available for 135 (90%). Because significant heterogeneity was observed (*P* < 0.00001; I^2^ = 98%), the random-effect model was employed. The results indicated that ARB did indeed reduce HOMA-IR (MD 1.29; 95% CI [0.13, 2.45]; *P* = 0.03; Figure 4).

**Figure 5:**
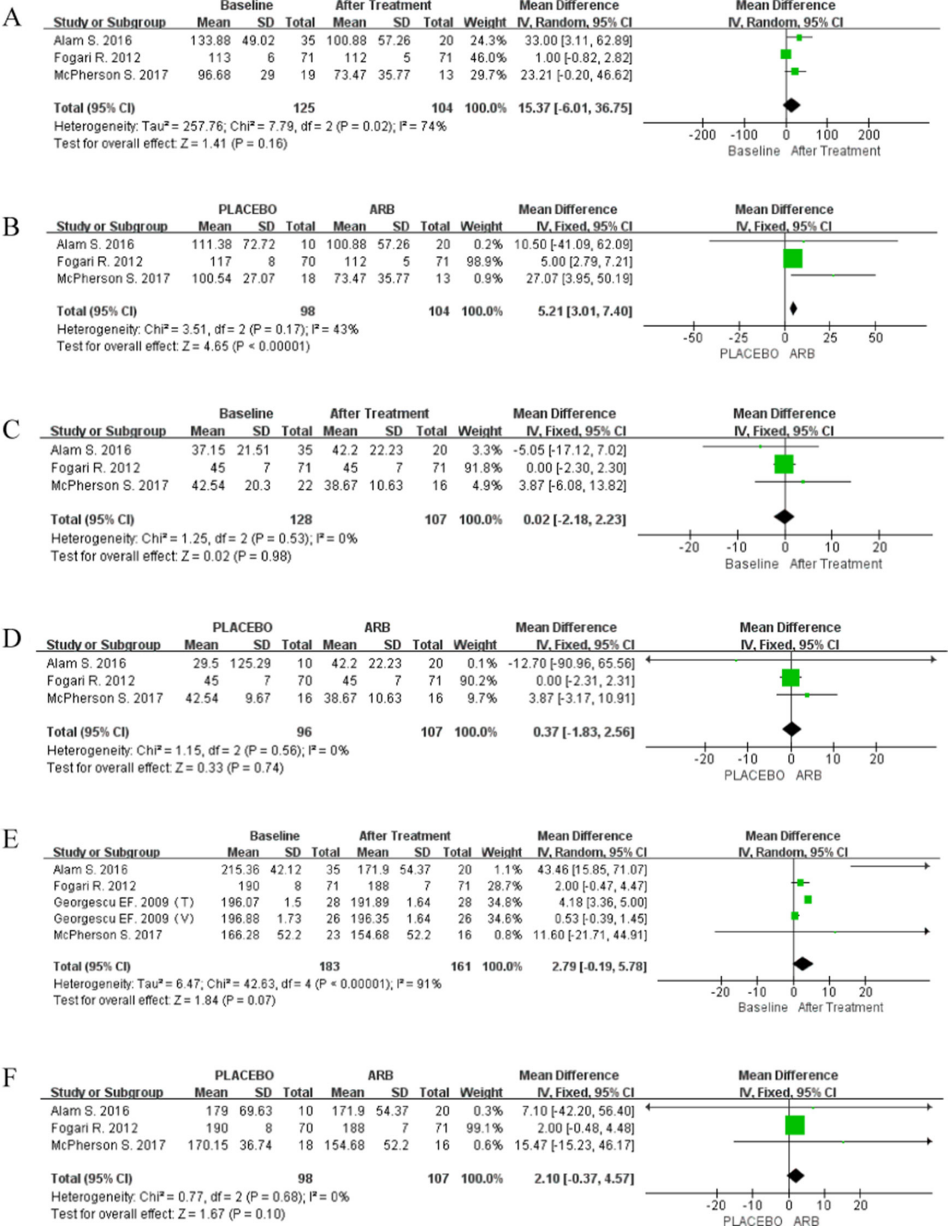
(**A**) Forest plot of the mean differences in the changes in LDL levels (single arm meta-analysis). (**B**) Forest plot of the mean differences in the changes in LDL levels (RCT-based meta-analysis). (**C**) Forest plot of the mean differences in the changes in HDL levels (single arm meta-analysis). (**D**) Forest plot of the mean differences in the changes in HDL levels (RCT-based meta-analysis). (**E**) Forest plot of the mean differences in the changes in TC levels (single arm meta-analysis). (**F**) Forest plot of the mean differences in the changes in TC levels (RCT-based meta-analysis).

Data for LDL levels were only reported in three RCTs, and the random-effect model was used for single-arm meta-analysis of these studies (*P* = 0.02; I^2^ = 74%). Despite the lack of statistical significance, ARB did decrease LDL levels (MD 15.37; 95% CI [-6.01, 36.75]; *P* = 0.16; Figure 5A). Furthermore, RCT-based meta-analysis did not reveal any evidence of heterogeneity (*P* = 0.17; I^2^ = 43%). Therefore, ARB treatment reduced LDL levels (MD 5.21; 95% CI [3.01, 7.40]; *P* < 0.00001; Figure 5B).

Data for HDL levels were available in three RCTs. The fixed-effect model was used because of low heterogeneity in both single-arm (*P* = 0.53; I^2^ = 0%) and RCT-based (*P* = 0.56; I^2^ = 0%) meta-analyses. However, no improvement in HDL levels was observed in response to ARBs in either meta-analysis (single-arm: MD 0.02; 95% CI [-2.18, 2.23]; *P* = 0.98 Figure 5C; RCT-based: MD 0.37; 95% CI [-1.83, 2.56]; *P* = 0.74 Figure 5D).

Four articles reported data on TC levels. The random-effect model was used in the single-arm meta-analysis (*P* < 0.00001; I^2^ = 91%), but the fixed-effect model was used in the RCT-based meta-analysis (*P* = 0.68; I^2^ = 0%). ARB appeared to reduce TC levels in both meta-analyses, albeit in a non-significant manner (single-arm: MD 2.79; 95% CI [-0.19, 5.78]; *P* < 0.00001; Figure 5E; RCT-based: MD 2.10; 95% CI [-0.37, 4.57]; *P* = 0.10; Figure 5F).

### Liver histology and degree of fibrosis

**Figure 6:**
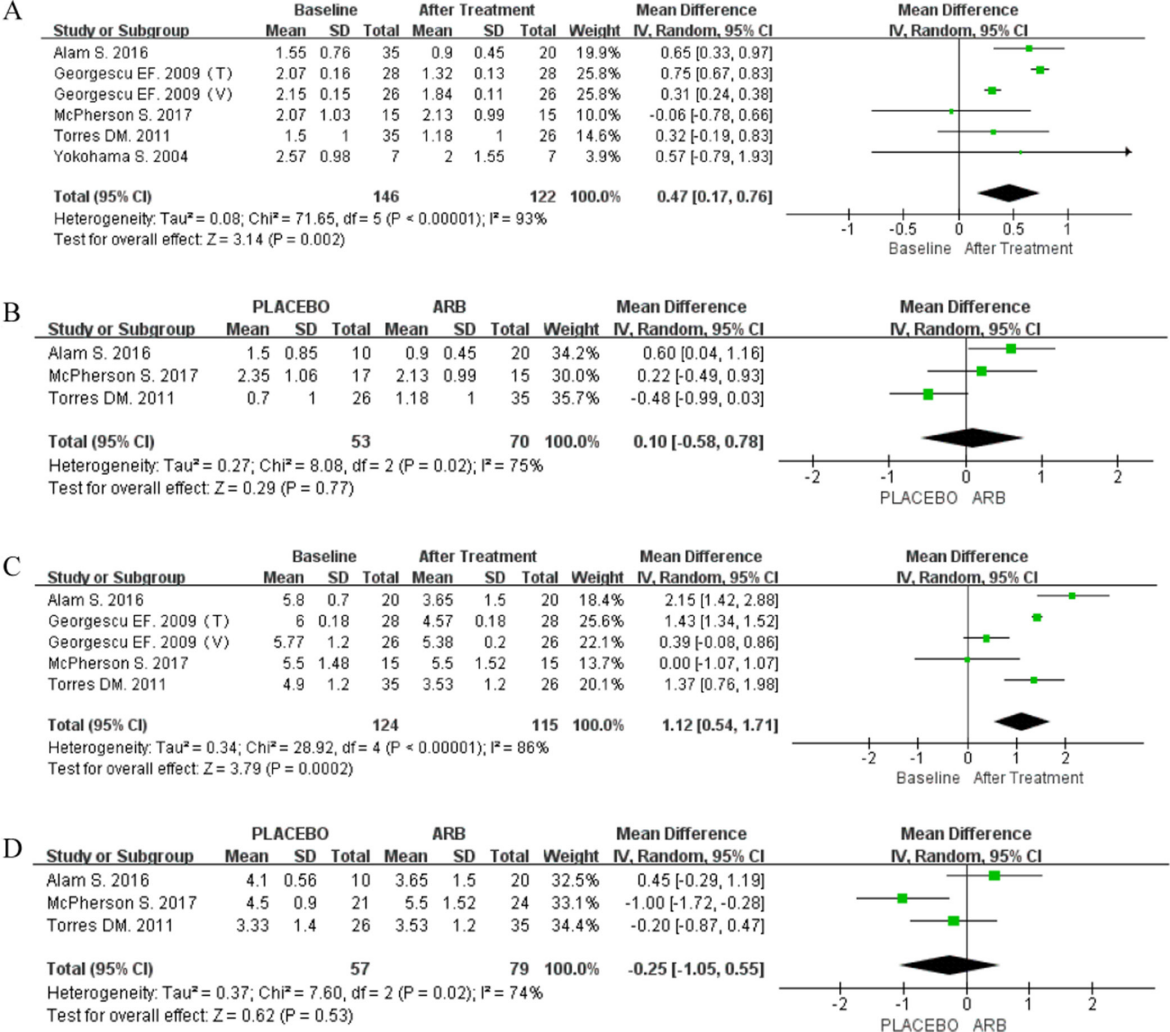
(**A**) Forest plot of the mean differences in the changes in F-S (single arm meta-analysis). (**B**) Forest plot of the mean differences in the changes in the F-S (RCT-based meta-analysis). (**C**) Forest plot of the mean differences in the changes in the NAS (single arm meta-analysis). (**D**) Forest plot of the mean differences in the changes in the NAS (RCT-based meta-analysis).

Data for the degree of fibrosis were pooled from five articles, including three RCTs. Single-arm meta-analysis using the random-effect model (*P* < 0.00001; I^2^ = 93%) showed a significant reduction in F-S in response to ARB treatment (MD 0.47; 95% CI [0.17, 0.76]; *P* = 0.002; Figure 6A). However, RCT-based meta-analysis using the random-effect model (*P* = 0.02; I^2^ = 75%) failed to reveal any significant difference in response to ARB treatment (MD 0.10; 95% CI [-0.58, 0.78]; *P* = 0.77; Figure 6B).

Four studies provided data for the NAS, including one single-arm clinical trial. Due to significant heterogeneity, a single-arm meta-analysis was performed using the random-effect model (*P* < 0.00001; I^2^ = 86%), and the results showed the NAS to be significantly reduced by ARB treatment (MD 1.12; 95% CI [0.54, 1.71]; *P* = 0.0002; Figure 6C). However, RCT-based meta-analysis using the random-effect model (*P* = 0.02; I^2^ = 74%) failed to show a significant improvement in the NAS by ARBs compared with the control treatment (MD -0.25; 95% CI [-1.05, 0.55; *P* = 0.53]; Figure 6D).

### Body mass index

**Figure 7:**
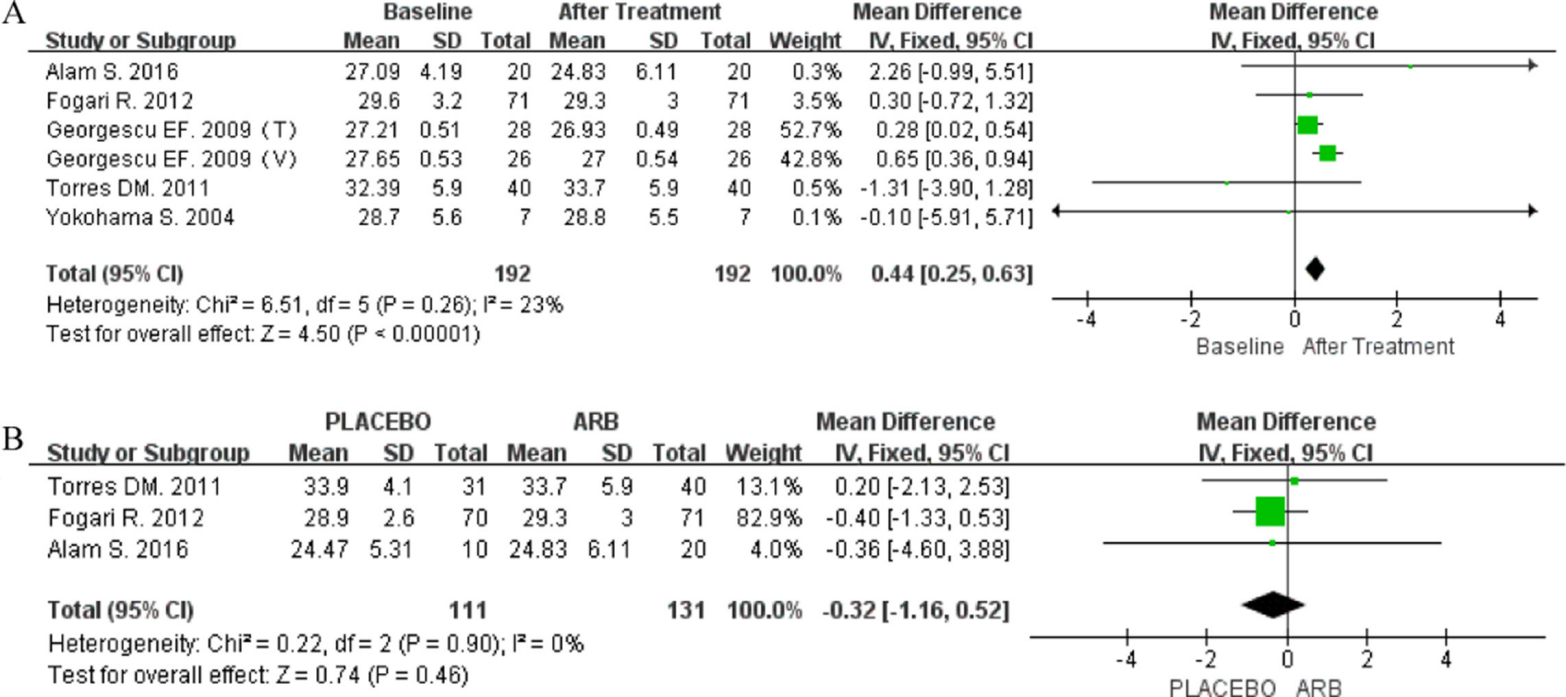
(**A**) Forest plot of the mean differences in the changes in BMI (RCT-based meta-analysis). (**B**) Forest plot of the mean differences in the changes in BMI (RCT-based meta-analysis).

Five articles included data on BMI post-treatment. The fixed-effect model was used in both single-arm (*P* = 0.26; I^2^ = 23%) and RCT-based (*P* = 0.90; I^2^ = 0%) meta-analyses. The former analysis indicated that BMI was significantly reduced following treatment with ARBs (MD 0.44; 95% CI [0.25, 0.63]; *P* < 0.00001; Figure 7A), whereas the latter did not show any significant difference in BMI in response to ARBs (MD -0.32; 95% CI [-1.16, 0.52]; *P* = 0.46; Figure 7B).

### Adverse events

Only three studies reported adverse events. The studies by McPherson et al. [24] (28419124) and Torres et al. [20] argued that the adverse events observed were independent of the ARB treatment [20, 24]. However, Alam et al. [22] reported several adverse events related to ARBs, including mild headache, dizziness and abdominal pain, though the authors did stipulate that the treatment did not need to be discontinued due to these adverse events. In general, ARBs have been widely used in clinical practice and are safe for NAFLD patients.

## DISCUSSION

The RAS is closely related to inflammation, oxidative stress and fibrosis in NAFLD, and angiotensin II (Ang II) is known to promote various cellular and molecular pathophysiological processes. Here, we attempt to assess the effects of ARBs on patients with NAFLD or NASH and to study whether ARBs can decrease hepatic histology scores and plasma liver enzyme activities as well as ameliorate histological changes.

### Summary of evidence

This systematic review includes results from RCT-based meta-analyses of four studies including 362 patients and single-arm meta-analysis of eight studies including 525 patients. To the best of our knowledge, this is the first RCT-based meta-analysis conducted to comprehensively evaluate the therapeutic effects of ARBs on patients with NAFLD. Our results support the use of ARBs as a practical and effective treatment strategy for patients with NAFLD, particularly because of the reductions in TC and LDL levels.

### Potential improvements in liver function

A small fraction of the RCTs included in this review reported significant differences between the ARB and placebo groups in terms of ALT levels. However, our pooled analysis demonstrated a clear downward trend in ALT levels, even though this decrease did not reach statistical significance. Consistent with our results from single-arm meta-analysis, a cross-sectional study of 290 hypertensive patients with NAFLD reported that AST levels were significantly lower in patients receiving ARBs than those in patients not treated with ARBs [4].

Telmisartan and valsartan are both commonly used in the clinic, and these drugs have been reported to improve ALT levels [21]. Forty-eight weeks of losartan treatment was also shown to significantly reduce serum aminotransferase levels in NASH patients [19].

### Improvements in lipometabolism and insulin resistance

IR is observed in approximately 95% of NAFLD patients [27] and is recognized as one of the pathophysiological hallmarks of NAFLD [28]. The therapeutic principles of NAFLD rely on targeting IR, which is pathologically linked to MS. Interestingly, previous studies have demonstrated a relationship between the RAAS and IR [29–31]. In hypertensive patients, ACEIs were reported to exert additive effects on lowering serum insulin concentrations and improving HOMA-IR scores. In non-modulating hypertensive patients, ARBs with partial PPAR-gamma agonist activity have also been shown to enhance insulin sensitivity [32, 33]. Moreover, the RAAS not only reduces blood pressure but also increases insulin sensitivity in patients with chronic kidney disease [4]. Nonetheless, our study adds to the accumulating evidence indicating that ARBs do not affect IR in patients with NAFLD. The lack of an effect on IR in our study may be due to the different diseases and different types of ARB used, such as telmisartan, valsartan [21]. Further studies are warranted to explore this issue.

Our RCT-based meta-analysis revealed lipid profile amelioration by ARBs, as indicated by the lower levels of plasma LDL and TC, consistent with previous findings [34–38]. Telmisartan has been reported to mildly improve the lipid profile [37]: after six months of telmisartan treatment (up to 80 mg/day), TC and LDL levels were found to be significantly reduced in hypertensive patients (*n* = 197), though HDL levels were not significantly altered. In the study by Derosa et al., patients with T2DM (*n* = 116) were treated with telmisartan (40 mg/day) [39] for 12 months, and improvements in TC (-9%; *P* < 0.01 vs baseline) and LDL (-11.5%; *P* < 0.01 vs baseline) levels were observed. In contrast to valsartan, which lacks lipid-lowering effects, the PPAR-gamma ligand effect of telmisartan enables it to ameliorate IR, and this likely contributes to its beneficial effects on the lipid profile [40].

Nevertheless, a study by Ichikawa et al. showed that telmisartan treatment did not affect, TC, HDL or LDL levels [33, 41, 42]. These differences in findings may be partly attributed to differences in patient groups, as most of the patients in the study by Ichikawa et al. had hypertension and T2DM but not NAFLD.

Based on our single-arm meta-analyses, we cautiously speculate that ARB treatment plus lifestyle modification may have a greater impact on lowering LDL levels than ARB treatment alone.

### Lack of improvements in liver histology and degree of fibrosis

Our pooled analysis showed a lack of beneficial effect of ARBs on fibrosis and NAS, which is consistent with the findings of McPherson et al., [24] despite the substantial amount of clinical data indicating that ARBs have a favorable effect on fibrosis. In the study by Orlic et al., patients treated with ACEIs or ARBs exhibited a significantly lower degree of liver stiffness, as assessed by transient elastography (TE) (Fibroscan^®^-CAP), than patients without ARB treatment [4]. However, no differences in steatosis or lobular inflammation were observed. Interestingly, a lower extent of ballooning and a lower NAS were observed in patients treated with ACEIs or ARBs than in untreated patients [4]. Moreover, after six months of losartan administration, fibrosis was ameliorated in patients with chronic hepatitis C [35, 43, 44]. In addition, losartan improved hepatic steatosis and serum biomarkers of fibrosis in patients with NASH [19, 21, 23]. Although RAS-B agents resulted in no differences in the grade of steatosis and lobular inflammation, a lower grade of ballooning, NAS index and stage of fibrosis were observed in hypertensive patients with biopsy-proven NAFLD [45]. However, another study on telmisartan (group T) and valsartan (group V) reported that only the former reduced steatosis and the NAS and F-S indices [21].

The main reason for the controversial results in the current meta-analysis may because of the small number of included studies. Histopathological examination is the gold standard for the diagnosis of NASH, but it is limited due to its invasive nature. Non-invasive tests such as ultrasonography and fibroscanning were performed for some of the included patients, which accounted for the fewer number of patients undergoing liver biopsy. Assessment of the degree of heterogeneity may not be reliable in such a small number of studies, and this may contribute to the lack of certainty in the results. Moreover, 7.1 years [46] are typically required for NASH to progress to another stage of fibrosis. An average 5-year period of observation should be considered, [47] and re-biopsy after this timeframe is supported by EASL-EASD-EASO guidelines [48]. Therefore, further studies conducted for longer durations are needed to examine the benefits of anti-fibrotics.

### Limitations

There are certain limitations of our study. Because only a handful of commonly established non-invasive biomarkers have been used in clinical trials of NAFLD/NASH, primary outcomes were assessed based on histological and biochemical markers such as liver enzymes, liver histology, degree of fibrosis and lack of invasion via TE. Results that are beyond the shared characteristics of the included study population cannot be generalized. Moreover, methodological differences and variable durations of treatment can lead to significant statistical heterogeneity, which cannot be solved by subgroup analysis. As negative results are less likely to be published, our search was confined in that regard as well. Due to the limited number of existing studies, it was difficult to perform publication bias analysis. The Cochrane handbook indicates that tests for funnel plot asymmetry should be used only when there are at least 10 studies included in the meta-analysis, as the power of the test is too low to distinguish chance from real asymmetry when studies are involved (http://handbook-5-1.cochrane.org/). Therefore, the results need to be treated with caution; analysis of existing clinical data may enable us to identify additional studies.

## CONCLUSIONS

We draw the following conclusion cautiously: although ARBs can significantly decrease plasma LDL and TC levels, current evidence is insufficient to support the efficacy of ARB in the management of fibrosis and HOMA-IR in NAFLD patients. Further clinical trials with larger sample sizes and longer follow-up durations are needed to strengthen the evidence for the efficacy of ARBs in treating NAFLD.

### Ethical considerations

As all reviews and analyses were based on previously published studies, no ethical approval was necessary.
